# Systematic review of structured care pathways in major depressive disorder and bipolar disorder

**DOI:** 10.1186/s12888-022-04379-z

**Published:** 2023-02-02

**Authors:** Helena Kyunghee Kim, Suman Banik, Muhammad Ishrat Husain, Victor Tang, Robert Levitan, Zafiris J. Daskalakis, Stefan Kloiber

**Affiliations:** 1 Department of Psychiatry, University of Toronto, Toronto, ON Canada; 2 Yorkville University, Fredericton, NB Canada; 3 Centre for Addiction and Mental Health, Campbell Family Mental Health Research Institute, 100 Stokes Street, Toronto, ON M6H 1J4 Canada; 4 Department of Psychiatry, University of California San Diego, San Diego, USA

**Keywords:** Structured care pathway, Major depressive disorder, Bipolar disorder, Systematic review, Treatment algorithm

## Abstract

**Background:**

Structured care pathways (SCPs) consist of treatment algorithms that patients advance through with the goal of achieving remission or response. These SCPs facilitate the application of current evidence and adequate treatment, which potentially benefit patients with mood disorders. The aim of this systematic review was to provide an updated synthesis of SCPs for the treatment of depressive disorders and bipolar disorder (BD).

**Method:**

PubMed, PsycINFO, and Embase were searched through June 2022 for peer-reviewed studies examining outcomes of SCPs. Eligibility criteria included being published in a peer-reviewed journal in the English language, reporting of intervention used in the SCP, and having quantitative outcomes. Studies Cochrane risk of bias tool was used to assess quality of RCTs.

**Results:**

Thirty-six studies including 15,032 patients were identified for qualitative synthesis. Six studies included patients with BD. The studies were highly heterogeneous in design, outcome measures, and algorithms. More than half of the studies reported superiority of SCPs over treatment as usual, suggesting that the standardized structure and consistent monitoring inherent in SCPs may be contributing to their effectiveness. We also found accumulating evidence supporting feasibility of SCPs in different settings, although dropout rates were generally higher in SCPs. The studies included were limited to being published in peer-reviewed journals in English language. The heterogeneity of studies did not allow quantitative evaluation.

**Conclusions:**

The findings of our study suggest that SCPs are equally or more effective than treatment as usual in depression and BD. Further studies are required to ascertain their effectiveness, particularly for BD, and to identify factors that influence their feasibility and success.

## Background

Major depressive disorder (MDD) and bipolar disorder (BD) are common mental health conditions worldwide that are associated with significant morbidity and mortality. The burden of these conditions spans across multiple domains, including functional, social, occupational, and overall quality of life [23, 36, 44, 73]. In addition to several years lost to disability, life expectancy is decreased by 7–11 years in patients with MDD and BD [14], which is not only caused by the 20-fold increased risk of suicide [16] in MDD, but also attributable to increased physical illness, such as comorbid metabolic and cardiovascular disorders [9, 17, 47].

While various treatments are available, including different modalities of psychotherapy, pharmacological treatments, and neurostimulation, approximately 1/3 of patients with MDD [22, 54, 67] show limited symptom improvement contributing to high individual suffering, healthcare costs and economic burden [40, 43]. Structured care pathways (SCPs) are an evidence-based treatment algorithm consisting of a series of steps to guide practitioners in the management of patients with a specific condition until symptom remission is achieved. SCPs have been gaining attention in the past two decades in many areas of medicine, including psychiatry [15, 24, 56]. They facilitate the development and implementation of tailored protocols based on ‘up-to-date’ evidence, capacity building, and sustainable change in care delivery to improve quality, safety and services. SCPs provide an optimal infrastructure to implement guidelines and quality standards, decrease unwanted, sub-standard variations in practice, and improve patient satisfaction [13]. Several treatment algorithms for patients with mood disorders with or without comorbidities have been proposed based on existing evidence. However, many of these algorithms have not been examined for their effectiveness or applicability in clinical trials [24, 25, 31, 33, 65].

The objective of this work was to provide an updated systematic review on the effectiveness of SCPs and treatment algorithms for treating individuals with MDD and BD.

## Methods

### Search methods

“Depression” was used to include major depressive episode, major depressive disorder, or depressive episode, depending on the terminology used by the studies included in this review. *Pubmed, EMBASE and PsycINFO* databases were searched up to June 18^th^, 2022 using the following search terms that included phrases and Boolean operators: “integrated care” AND depression, “treatment algorithm” AND depression, “measurement based” AND depression, “stepped care” AND depression, “integrated care” AND bipolar, “treatment algorithm” AND bipolar, “measurement based” AND bipolar, “stepped care” AND bipolar. Searches and initial screening were performed by HKK and SB independently, with consultation and review by SK. This review protocol was not registered. Instead, the search terms, inclusion / exclusion criteria, protocol for literature search, data extraction, and quality assessment were established, and approved by the senior author prior to commencing the systematic review.

### Eligibility criteria

For our eligibility criteria, we defined SCP as a treatment algorithm, outline or program containing serial intervention components including pharmacological, psychological, and/or neurostimulation interventions with patients advancing through the program depending on their improvement or deterioration in symptoms with the aim of achieving treatment response and/or remission. The eligibility criteria for the studies were: a) published in a peer-reviewed journal, b) published in the English language, c) designed with the aim of quantitatively measuring treatment outcomes of SCPs and reports on the outcomes, d) includes or addresses patients with depressive disorders (including major depressive episode, major depressive disorder, or depressive episode, depending on the terminology used by the authors; Table 1), and/or bipolar disorder (regardless of if patients are in manic/ depressive episode), e) includes specific information on the psychotherapy/pharmacotherapy/neurostimulation interventions included in the algorithm (i.e., does not simply state that a patient will receive medications or therapy without specifying the type/class), f) treatment algorithm is implemented in a medical center that can administer all interventions in the algorithm (i.e. SCP was not a tool to stratify who gets referred to tertiary centers or physicians), and h) implemented or proposed care plan fits the definition of SCP described above. Clinical trials that were not designed with the aim of evaluating a treatment algorithm or a SCP, such as EMBARC (https://clinicaltrials.gov/ct2/show/NCT01407094) or CAN-BIND (https://clinicaltrials.gov/ct2/show/NCT04162522) were not included, since their stated aim was to identify disease / predictive / moderating / mediator markers in depression.

**Table 1:** Summary of structured care pathways for major depressive disorder/ depression (A) and bipolar disorder (B). Names of medication class and psychotherapy have been bolded. Dosages written, unless the range is specified, indicate maximum dosages

**A. Major depressive disorder**		
	Intervention (reference, study design)	
Scale/assessment used for measurement-based care / treatment algorithm decisions		1. BDI ([26], CS; [27], OBS; [69], OBS) 2. BRMS ([51], RCT; [7], RCT; [1], OBS) 3. CGI ([21], CS; Ribeiz [50]; OBS), CGI-B subscale (Agid [2], OBS) 4. GAD-7 ([61], RCT) 5. HAMD (Ribeiz [50]; OBS) -21items ([52], RCT), HAMD ([4], RCT; [12], RCT), -17 items ([10], OBS; [72], OBS) 6. IDS-C (Kurian [37], NRCT; [66], CS) 7. MADRS ([11], OBS; Ribeiz [50]; OBS) 8. PHQ-9 ([70], RCT; [61], RCT; [35], RCT; [20], RCT; [18], RCT) 9. QIDS-SR ([29], RCT; [56], CS; [6], CS; [60], NRCT; [57], OBS)
Treatment algorithm steps: Step 1	Pharmacotherapy	**Discontinuation of previous medication (**[51], RCT; [7], RCT; [1], OBS)
		**Monotherapy**
		**Any antidepressant** ([52], RCT; [70], RCT)
		**SSRIs**
		1. SSRIs in general (Kurian [37], NRCT; [66], CS; Ribeiz [50], OBS)
		2. Citalopram ([60], NRCT; [21], CS for childhood), 30 mg ([4], RCT; [12], RCT)
		3. Escitalopram ([35], RCT; [20], RCT)
		4. Paroxetine ([21], CS in childhood; [46], OBS – with advanced cancer, moderate-severe depression), 20 mg ([29], RCT; [11], OBS; Agid [2], OBS)
		5. Sertraline ([21], CS for childhood; RIbeiz [50], OBS; [35], RCT; [20], RCT), 50 mg (Turner-Sokes [69], OBS with brain injury)
		6. Fluvoxamine ([10], OBS)
		7. Fluoxetine ([21], CS – for childhood), 20 mg (Agid [2], OBS),
		**TCAs**
		1. TCAs in general (Kurian [37], NRCT)
		2. Amitriptyline or clomipramine ([46], OBS – with advanced cancer and no oral intake; [3], OBS – advanced cancer)
		3. Imipramine ([10], OBS; [2, 3], OBS – advanced cancer; [72], OBS)
		4. Amoxapine ([46], OBS – with advanced cancer, moderate-severe)
		**SNRIs**
		1. Venlafaxine (Kurian [37], NRCT; [66], CS; [72], OBS; [35], RCT; Ell [20], RCT)
		2. Milnacipran ([46], OBS – with advanced cancer, moderate-severe)
		**Benzodiazepines**
		1. Any benzodiazepine ([3], OBS – with advanced cancer and disturbed oral intake)
		2. Alprazolam ([46], OBS – with advanced cancer, mild depression)
		3. Diazepam or bromazepam ([3], OBS – with advanced cancer and disturbed oral intake)
		**Other**
		1. Bupropion (Kurian [37], NRCT; [66], CS; [35], RCT; [20], RCT)
		2. Mirtazapine (Kurian [37], NRCT; Ribeiz [50], OBS; [35], RCT; [20], RCT), 30 mg ([29], RCT)
		3. Nefazodone (Kurian [37], NRCT; [66], CS)
		4. Mianserin ([46], OBS – with advanced cancer, moderate-severe)
		5. Tandospirone ([3], OBS – with advanced cancer and normal oral intake)
		6. Methylphenidate ([46], OBS – with advanced cancer, mild depression; [3], OBS – with advanced cancer)
		7. Sulpiride or hydroxyzine ([3], OBS – with advanced cancer and disturbed oral intake)
		**Augmentation/ combination therapy**
		**Antipsychotic**
		1. Typical antipsychotic, olanzapine/ risperidone if psychotic depression (Kurian [37], NRCT; [66], CS)
		**Antidepressant**
		1. Amoxapine if psychotic depression (Kurian [37], NRCT; [66], CS) Sertraline + naltrexone for MDD + AUD ([56], CS; [6], CS; [57], OBS)
	Psychotherapy/ behavioral interventions	**Psychotherapy**
		1. PST-PC ([70], RCT)
		2. PST ([35], RCT; [20], RCT)
		3. IPT ([4], RCT)
		4. Brief-psychotherapy 8 sessions ([26], CS; [27], OBS)
		5. Telephone-guided computerized CBT ([18], RCT)
		**Counselling/ psychoeducation**
		1. Psychoeducation ([26], CS; [27], OBS; [41], OBS; [42], CS – for mild MDD) – 4 week course ([61], RCT)
		2. Self-help or counselling ([18], RCT; [26], CS; [27], OBS; [41], OBS; [42], CS)
	Neurostimulation	None
Step 2	Pharmacotherapy	**Monotherapy**
		1. Start antidepressant monotherapy ([26], CS; [27], OBS; [1], OBS; [42], CS – for moderate to severe MDD)
		2. Dose escalation ([52], RCT; [4], RCT; [12], RCT; [29], RCT; [56, 69], OBS – with brain injury; [11], OBS; Agid [2], OBS; [35], RCT; [20], RCT)
		3. Switch to a different antidepressant ([52], RCT; [70], RCT; [56], CS; [21], CS – for childhood)
		**SSRIs**
		1. Sertraline ([60], NRCT)
		2. Fluoxetine ([56], CS; [6], CS; [57], OBS)
		3. Paroxetine ([46], OBS – with advanced cancer)
		**TCAs**
		1. Amitriptyline or nortriptyline ([46], OBS – with advanced cancer; [3], OBS – with advanced cancer)
		2. Clomipramine or imipramine ([3], OBS – with advanced cancer)
		3. Amoxapine ([46], OBS – with advanced cancer),
		4. Switch from non-TCA to TCA or vice versa (Kurian [37], NRCT; [66], CS – for psychotic depression)
		**SNRIs**
		1. Venlafaxine XR ([60], NRCT; [10], OBS; [57], OBS)
		2. Milnacipran ([46], OBS – with advanced cancer)
		**Others**
		1. Bupropion SR ([60], NRCT)
		2. Mianserin ([46], OBS – with advanced cancer),
		3. Mirtazapine ([57], OBS)
		**Augmentation/ combination therapy**
		**Mood stabilizers**
		1. Lithium ([52], RCT; [10], OBS; Kurian [37], NRCT; [66], CS; Ribeiz [50], OBS; [72], OBS)
		2. Valproic acid augmentation (Ribeiz [50], OBS)
		**Others**
		1. Combination of citalopram + bupropion SR ([60], NRCT)
		2. Thyroid hormone (Kurian [37], NRCT; [66], CS; Agid [2], OBS)
		3. Buspirone (Kurian [37], NRCT; [66], CS; [60], NRCT)
	Psychotherapy/ behavioral interventions	**Counselling/ psychoeducation**
		1. Counselling ([41], OBS)
		2. Person-centered experiential counselling ([18], RCT)
		**Psychotherapy**
		1. PST-PC ([70], RCT)
		2. PST ([42], CS – mild MDD; [35], RCT; [20], RCT)
		3. 10-week coping with depression/ anxiety course ([61], RCT)
		4. CBT ([18], RCT; [60] – NRCT; [26], CS; [27], OBS; [42], CS – for moderate to severe MD)
		5. Brief therapy ([42], CS – mild MDD; [41], OBS)
		6. Group therapy or IPT ([26], CS; [27], OBS; [42], CS – for moderate to severe MDD)
		7. Brief psychodynamic therapy ([42], CS – for moderate to severe MDD)
		**Behavioral intervention**
		1. Sleep deprivation ([51], RCT; [7], RCT)
	Neurostimulation	None
Step 3	Pharmacotherapy	**Monotherapy (switch or start)**
		**Any antidepressant** ([51], RCT; [7], RCT; [41], OBS)
		**TCAs**
		1. Nortriptyline ([60], NRCT)
		2. Clomipramine ([10], OBS; Agid [2], OBS)
		**MAOIs**
		1. Tranylcypromine ([52], RCT)
		2. Phenelzine ([10], OBS)
		**Others**
		1. Mirtazapine ([56], CS; [6], CS; [60], NRCT)
		2. Venlafaxine ([56], CS; Agid [2], OBS)
		3. Bupropion SR 200–400 mg/ day in two divided doses ([4], RCT; [12], RCT)
		**Augmentation/ combination therapy**
		**Specific agents:**
		1. Lithium (Kurian [37], NRCT; [60], NRCT; [66], CS; [1], OBS)
		2. T3 ([52], RCT; [60], NRCT)
		3. Bupropion SR 200–400 mg/day in two divided doses if partial response to antidepressant ([4], RCT; [12], RCT)
		**Others:**
		1. Antidepressant combination ([61], RCT; [70], RCT; [35], RCT; [20], RCT; [21], CS – for childhood)
		2. Complex polypharmacy/ combination therapy (Ribeiz [50], OBS; [42], CS – for moderate to severe MDD)
	Psychotherapy/ behavioral intervention	**Switch to psychotherapy only**
		1. PST-PC ([70], RCT)
		**Augment with psychotherapy**
		1. CBT or IPT ([41], OBS; [42], CS)
		2. Behavioral therapy or brief psychodynamic therapy ([42], CS)
		3. Depression/ anxiety course ([61], RCT)
		4. PST ([35], RCT; [20], RCT)
	Neurostimulation	ECT ([52], RCT; for psychotic depression – Kurian [37], NRCT; [66], CS; Ribeiz [50], OBS; [72], OBS)
Step 4	Pharmacotherapy	**Monotherapy**
		1. Dose-escalation ([51], RCT; [7], RCT)
		2. Venlafaxine ([21], CS – for childhood), 150–300 mg qAM ([4], RCT; [12], RCT)
		3. Nefazodone, bupropion or mirtazapine ([21], CS – for childhood)
		4. Tranylcypromine ([60], NRCT; [1], OBS)
		**Augmentation/ combination therapy**
		**Lithium**
		1. Lithium augmentation with a previously untried antidepressant for psychotic depression (Kurian [37], NRCT; [66], CS)
		2. Clomipramine + lithium ([10], OBS)
		**Others**
		1. TCA + SSRI (Kurian [37], NRCT; [66], CS)
		2. Bupropion SR + SSRI (Kurian [37], NRCT; [66], CS)
		3. Nefazodone + SSRI (Kurian [37], NRCT; [66], CS)
		4. Bupropion SR + nefazodone (Kurian [37], NRCT; [66], CS)
		5. Venlafaxine XR + mirtazapine ([60], NRCT)
		6. Augment with nortriptyline to plasma concentration of 80–120 ng/mL if partial response to existing SSRI or SNRI ([4], RCT; [12], RCT)
	Psychotherapy/ behavioral intervention	1. Combination of psychotherapy and pharmacotherapy ([41], OBS) 2. Light therapy ([41], OBS)
	Neurostimulation	ECT ([10], OBS)
Step 5	Pharmacotherapy	**Monotherapy**
		1. Nortriptyline to plasma concentration 80–120 ng/mL if no response to antidepressant ([4], RCT; [12], RCT)
		**Augmentation/ combination therapy**
		1. Lithium ([51], RCT; [7], RCT), to plasma concentration of 0.6–0.8 mEq/L ([4], RCT; [12], RCT)
	Psychotherapy/ behavioral intervention	None
	Neurostimulation	ECT (Kurian [37], NRCT; [41], OBS; [1], OBS)
Step 6	Pharmacotherapy	**Monotherapy**
		**Antidepressants**
		1. Mirtazapine 30–45 mg qhs if no response to antidepressant ([4], RCT; [12], RCT)
		2. Fluvoxamine (Kurian [37], NRCT)
		**Mood stabilizers**
		1. Lithium monotherapy ([51], RCT; [7], RCT)
		2. Lamotrigine monotherapy (Kurian [37], NRCT)
		**Antipsychotics**
		1. Olanzapine (Kurian [37], NRCT)
		**Combination therapy**
		1. Mirtazapine + bupropion (Kurian [37], NRCT)
	Psychotherapy/ behavioral intervention	None
	Neurostimulation	None
Step 7	Pharmacotherapy	MAOI and lithium combination therapy ([51], RCT; [7], RCT)
	Psychotherapy/ behavioral intervention	None
	Neurostimulation	None
Step 8	Pharmacotherapy	None
	Psychotherapy/ behavioral intervention	None
	Neurostimulation	ECT after discontinuation of medications ([51], RCT; [7], RCT)
**B. Bipolar disorder**		
St﻿eps	Intervention (reference, study design)	
Scale/assessment used for measurement-based care / treatment algorithm decisions	1. CGI-BP-I ([49], CS – for childhood) 2. BPRS ([62], CS; [64], OBS; [63], OBS) 3. BRMS ([7], RCT) – for bipolar depression 4. YMRS ([58],OBS – for childhood)	
Treatment algorithm steps: Step 1	Pharmacotherapy	**Discontinuation of previous medications** ([7], RCT – for DE) including destabilizing agents, antidepressants, stimulants and GABA-ergic agents ([58], OBS – for children)
		**Monotherapy**
		**Mood stabilizers**
		1. Mood stabilizer monotherapy for manic/mixed/ hypomanic episode ([49], CS – for childhood; [63], OBS)
		2. Carbamazepine or DVP if mixed or cycling ([62], CS; [64], OBS)
		3. DVP or lithium if euphoric ([62], CS; [64], OBS)
		**Antipsychotics**
		1. SGA monotherapy for prominent irritability without psychosis ([49], CS – for childhood)
		**Augmentation/ combination therapy**
		1. Mood stabilizer + SGA for manic/ mixed episode ([49], CS—for childhood)
		2. Mood stabilizer + bupropion SR or SSRI combination therapy for DE ([62], CS; [64], OBS)
	Psychotherapy/ behavioral intervention	None
	Neurostimulation	None
Step 2	Pharmacotherapy	**Monotherapy**
		**Mood stabilizers**
		1. Lithium, VPA ([58], OBS – for children), DVP ([63], OBS – if not psychotic)
		2. Switch to a different mood stabilizer ([49], CS – for childhood)
		**Antipsychotics**
		1. Antipsychotic ([63], OBS – if psychotic)
		2. SGA ([58], OBS – for children)
		**Augmentation/ combination therapy**
		1. Switch to another antidepressant (bupropion SR, SSRI, venlafaxine, nefazodone) and maintain mood stabilizer for DE ([62], CS; [64], OBS)
		2. Add lithium to existing mood stabilizer for mania/ hypomania ([62], CS; [64], OBS)
	Psychotherapy/ behavioral intervention	Sleep deprivation ([7], RCT – for DE)
	Neurostimulation	None
Step 3	Pharmacotherapy	**Monotherapy**
		1. Antidepressant ([7], RCT – for DE)
		2. Fluoxetine if depressed ([63], OBS)
		**Augmentation/ combination therapy**
		1. One or two mood stabilizers ([58], OBS – for children)
		2. DVP + carbamazepine combination therapy for mania/ hypomania ([62], CS; [64], OBS)
		3. Mood stabilizer and MAOI combination therapy for DE ([62], CS; [64], OBS)
	Psychotherapy/ behavioral therapy	None
	Neurostimulation	None
Step 4	Pharmacotherapy	**Monotherapy**
		1. Antidepressant dose-escalation ([7], RCT – for DE)
		**Augmentation/ combination therapy**
		1. Add SGA to existing mood stabilizer(s) for mania/ hypomania ([62], CS; [64], OBS)
	Psychotherapy/ behavioral intervention	None
	Neurostimulation	ECT for DE ([62], CS; [64], OBS)
Step 5	Pharmacotherapy	**Monotherapy**
		1. Lamotrigine for DE ([62], CS; [64], OBS)
		**Augmentation/ combination therapy**
		1. Lithium augmentation for DE ([7], RCT – for DE)
	Psychotherapy/ behavioral intervention	None
	Neurostimulation	ECT for mania/ hypomania ([62], CS; [64], OBS)
Step 6	Pharmacotherapy	1. Lamotrigine/ gabapentin for mania/ hypomania ([62], CS; [64], OBS)
		2. Lithium monotherapy for DE ([7], RCT)
	Psychotherapy/ behavioral intervention	None
	Neurostimulation	None
Step 7	Pharmacotherapy	Lithium and MAOI combination therapy for DE ([7], RCT)
	Psychotherapy/ behavioral intervention	None
	Neurostimulation	None
Step 8	Pharmacotherapy	None
	Psychotherapy/ behavioral intervention	None
	Neurostimulation	ECT ([7], RCT – for DE)

*Abbreviations*: *BDI* Beck depression inventory, *BPRS* Brief psychiatric rating scale, *BRMS* Bech-Rafaelson melancholia scale, *CBT* Cognitive behavioral therapy, *CGI* Clinical global impression scale, *CGI-BP-I* Clinical global impression scale for bipolar illness – improvement scale, *CS* Cohort study, *DE* Depressive episode, *DVP* Divalproex, *GAD-7* Generalized anxiety disorder 7-item scale, *HAMD* Hamilton depression rating scale, *IDS-C* Inventory of depressive symptomatology scale, clinician assessment, *IPT* Interpersonal psychotherapy, *MADRS* Montgomery-Asberg depression rating scale, *MAOI* Monoamine oxidase inhibitor, *NRCT* Non-randomized controlled trial, *OBS* Observational study without control group, *PHQ-9* Patient health questionnaire-9, *PST-PC* Problem-solving treatment in primary care, *qAM* Every morning, *qhs* Every evening, *QIDS-SR* Quick inventory of depressive symptomatology – self report, *RCT* Randomized clinical trial, *SGA* Second generation antipsychotics, *SNRI* Serotonin norepinephrine reuptake inhibitor, *SSRI* Selective serotonin reuptake inhibitor, *SCP* Structured care pathway, *T3* Triiodothyronine, *YMRS* Young mania rating scale, *VPA* Valproic acid

In this review, we included randomized controlled trials (RCTs) as well as cohort studies and observational studies without control groups to obtain results from a wider variety of settings and populations that are more reflective of everyday clinical practice [24].

### Data extraction and quality assessment

Study design, duration, number of subjects, algorithm used and its name, if available, control group, outcome measures and main outcomes (as defined by the original authors), dropout rates, and adverse events were extracted. Data extraction was performed by HKK and SB independently, with consultation and review by SK. For RCTs, non-randomized controlled trials, and cohort studies, author (year), number of participants in the SCP group and the comparator group, proportion of females, study duration and/or enrollment length, psychiatric condition treated, comparator group, main outcome measure, main result (SCP as effective as comparator or SCP greater/less effective than comparator), dropout rate for SCP and comparator (or both combined, depending on how it is reported in the study), and adverse events were reported. For RCTs only, the Cochrane Risk of Bias tool [30] was used to assess the quality of the studies based on the presence and quality of random sequence generation, allocation concealment, blinding of participants and outcome assessors, as well as potential for attrition bias, reporting bias, and other biases as identified. Quality assessment was performed by HKK and reviewed by SK. For observational studies without a control group, author (year), proportion of females, study duration and/or enrollment length, psychiatric condition treated, main outcome measure and main outcome as reported in original study, dropout rate, and adverse events were reported.

Summary of study characteristics, including the type of study (RCT, non-randomized controlled trial, cohort study, or observational study), number of participants, type of mood disorder studied (depression and/or bipolar disorder), study population (age group and comorbid condition, proportion of female participants), and number of steps of the SCP were synthesized and presented as a range. Studies were then stratified according to study type (RCT, non-randomized controlled trial, cohort study, or observational study), and comparator group, if applicable, study duration, main outcome measure, main outcomes, and dropout and adverse events were reported as ranges (e.g., for duration) or numbers of studies (e.g., for number of studies where dropout rates are higher in SCP compared to comparator group). Regardless of the study type, results were reported “as is” without any assumptions. If an item was not reported in the original manuscript, it was reported as not documented (ND).

We decided to provide a qualitative summary of studies examining SCPs in mood disorders, which is consistent with our aim of providing an updated review, rather than quantitatively synthesizing their effectiveness against comparators to provide a clinical recommendation.

## Results

### Selection of included studies

**Fig. 1:**
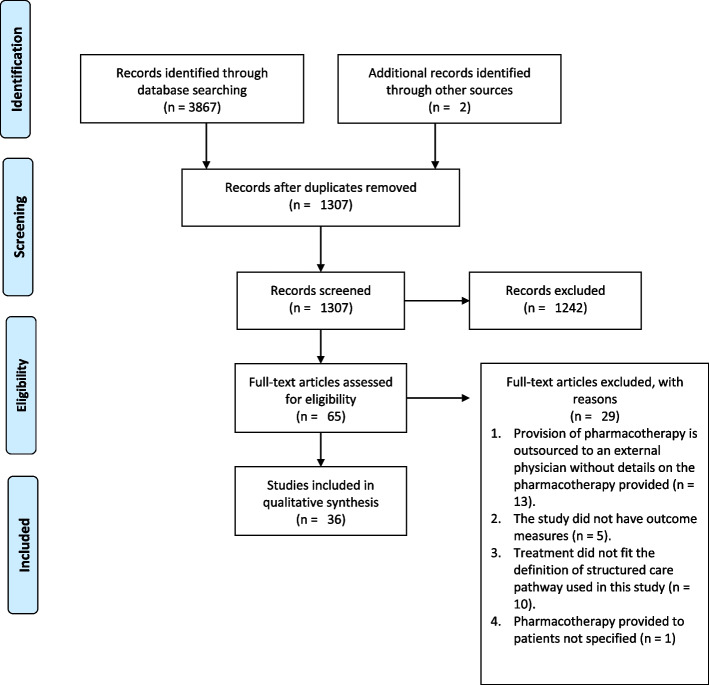
Preferred Reporting Items for Systematic Reviews and Meta-Analyses (PRISMA) flow diagram

PRISMA flow diagram [48] can be found in Fig. 1. Our search terms returned 3867 results. Two articles were added from a review of the included articles. One thousand three hundred seven articles remained after removing duplicates that were either generated by different databases or different search terms, of which 65 full-text articles were reviewed for eligibility. Of these articles, 29 articles were excluded for not meeting inclusion criteria described above (Fig. 1), resulting in inclusion of 36 studies for qualitative synthesis.

### Summary of structured care pathways for depression

A summary of care pathways for patients with depression is shown in Table 1A. Nine different scales were used to decide if patients should advance to the next stage of the pathway, with the HAMD [4, 10, 12, 50, 52, 72] and QIDS-SR [6, 29, 56, 57, 60] being the most common scales. The number of steps in a SCP ranged from 2 to 8.

As first step of SCPs, antidepressant monotherapy with a selective serotonin reuptake inhibitor (SSRI) was most consistently used, including citalopram, escitalopram, paroxetine, and sertraline [2, 4, 11, 12, 20, 21, 29, 35, 37, 50, 52, 60, 66, 69, 70], although other classes of antidepressants, including venlafaxine, bupropion, mirtazapine, and tricyclic antidepressants were also used in 9 SCPs [3, 10, 20, 35, 37, 46, 62, 66, 72]. Psychoeducation, self-help or counselling were suggested as first step in 6 studies [18, 26, 27, 41, 42, 61]. Psychotherapies applied in the first step of SCPs included problem solving therapy (PST) [20, 35, 70], interpersonal psychotherapy (IPT) [4] and brief-psychotherapy [26, 27], with other modalities such as cognitive behavioral therapy (CBT) being included in subsequent steps [18, 26, 27, 42, 60]. SCPs for patients with depression and psychiatric or medical comorbidities also included medications that are specific to these populations in the first step. These included naltrexone for patients with comorbid AUD [6, 56, 57] as well as stimulants and benzodiazepines for patients with advanced cancer [3, 46]. Subsequent steps of the SCPs involved combination treatment or dose escalation, starting a different antidepressant, combining with another antidepressant, or augmenting with a mood stabilizer, T3, an antipsychotic, or psychotherapy, with studies varying widely in the presence or absence of some of these options or the order in which they were integrated. Mood stabilizers such as lithium or valproic acid [1, 4, 7, 10, 12, 37, 50–52, 60, 66, 72], T3 [2, 37, 52, 60, 66], and monoamine oxidase inhibitors (MAOIs) [1, 7, 10, 51, 52, 60] were included in the later stages of SCPs. Ten studies included ECT as the last step in the SCP [1, 7, 10, 37, 41, 50–52, 66, 72].

### Summary of structured care pathways for bipolar disorder (BD)

Care pathways for patients with BD are summarized in Table 1B. Four scales were used to determine how patients proceeded through the SCP, with brief psychiatric rating scale (BPRS) being most commonly used by the three TMAP studies [62–64]. SCPs for the treatment of BD had between 3 to 8 steps.

The majority of SCPs included monotherapy with a mood stabilizer as first step [49, 62–64] of the algorithms, while combining a mood stabilizer with an antidepressant was also used as first step for depressive episodes [62, 64]. Subsequent steps involved a combination of switching to a different mood stabilizer, combining two mood stabilizers, or adding atypical antipsychotics or antidepressants to a mood stabilizer with variability in the amount of options offered as well as variability of the order. ECT was included as the last step in 3 studies [7, 62, 64].

### Summary of study characteristics

Eleven randomized controlled trials [4, 7, 12, 18, 20, 29, 35, 51, 52, 61, 70], 2 non-randomized clinical trials [37, 60], 8 cohort studies [6, 21, 26, 42, 49, 56, 62, 66], and 15 observational studies without control groups [1–3, 10, 11, 27, 41, 46, 50, 57, 58, 63, 64, 68, 72] were included using our inclusion criteria, which included 15,032 participants in total. Of these, 6 studies examined patients with BD [7, 49, 58, 62–64], where one study included both patients with MDD and bipolar depression [7]. Number of patients ranged from 15 to 3956 per group. While the majority of studies examined adults in the age range of 18 – 65 years, 4 studies examined older adults (≥ 60 years of age) [4, 12, 50, 70] and 3 studies examined youth up to 17 years old [21, 49, 58]. For articles that reported the number of female participants, the percentage ranged from 33 to 87%. Several studies examined comorbid conditions with depression or BD, including chronic diseases (i.e., diabetes, asthma, or COPD [61], alcohol use disorder/ dependence [6, 56, 57], anxiety [35], ADHD [21], complex disabilities [69], acute coronary syndromes [35], and advanced cancer [3, 20, 46]. The study length ranged from 4 weeks to 5 years, and the number of steps in a SCP ranged from 2 to 8 steps.

Two studies were from the Prevention of Suicide in Primary Care Elderly: Collaborative Trial (PROSPECT) [4, 12] and three studies were from the German Algorithm Project (GAP) [7, 51, 52]. Three studies were a part of the Improving Mood-Promoting Access to Collaborative Treatment (IMPACT) project or used its algorithm [20, 35, 70]. Five studies were from the Texas Medication Algorithm Project (TMAP), or its equivalent for children [21, 37, 62, 64, 66], and 3 studies were from the Depression and Alcoholism: Validation of an Integrated Care Initiative (DA VINCI) project or used its algorithm [6, 56, 57]. One study was from the Duke Somatic Algorithm Treatment for Geriatric Depression (STAGED) project [50], and one study used an adaptation of the Systematic Treatment Enhancement Program for Bipolar Disorder (STEP-BP) algorithm [58]. Sinyor and colleagues’ study [60] reviewed the clinical effectiveness outcomes of the 4 levels of the STAR*D trial, which was designed to examine the effectiveness of a treatment algorithm [28]. We included this article as it evaluated the treatment algorithm as a whole, which met our definition of a SCP, in that patients who did not remit were entered into the next level of the trial designed to improve treatment response [60].

### Randomized controlled trials

Randomized controlled trials (RCTs) are summarized in Table 2A. One RCT examined patients with bipolar depression as well as patients with unipolar depression [7], while other studies examined patients with MDD, dysthymia, or other depressive disorders. Treatment as usual (TAU), which is a term used to describe treatment that is delivered in accordance with routine care practices in that particular institution or care setting, was the comparator for all but one study [18]. Study length ranged from 12 weeks to 3 years, with one study being patient dependent [52]. For RCTs, the Hamilton depression rating scale (HAMD) with 17, 21 or 24 items was most commonly used as the outcome measure, with HAMD score reduction of ≥ 50% being the criterion for response in all 4 studies [4, 12, 29, 52]. There was greater variation in the cutoffs for remission, ranging from HAMD score less than 7 to less than 10. The Bech-Rafaelson Melancholia Rating Scale (BRMS) score less than 8 was also used as a cutoff for remission in two studies [7, 51]. The patient health questionnaire (PHQ-9), hospital anxiety and depression scale – anxiety (HADS-A), and symptom checklist 20 (SCL-20) were also used to measure treatment outcomes [18, 20, 35, 61, 70].

**Table 2:** Summary of randomized controlled trials (A), non-randomized controlled trials (B) and cohort studies (C) in patients with depressive disorders (MDD, MDE or other depressive disorder) or bipolar disorder

**Author (year)**	**N/ %F**	**Duration (study duration and/or enrollment length)**	**Condition**	**Comparator**	**Main outcomes**	**Dropout and adverse events**
**A. Randomized Controlled Trials**						
Alexopoulos [4]	SCP: 320 TAU: 279 %F: 71.6%	2y (study duration/ enrollment length)	Depression (older adults)	TAU	Suicidal ideation (Scale for suicide ideation) reduction SCP > TAU Remission (HAMD-24 < 7) SCP > TAU	Dropout SCP (43%) > TAU (37%). Adverse events ND
Bauer [7]	SCP: 74 TAU: 74 %F: 57% in SCP, 62% in TAU	12w (study duration/ enrollment length)	MDE, dysthymia, longer depressive reaction, and bipolar depression	TAU	Time to remission (BRMS < 8) SCP < TAU. Remission SCP = TAU	Dropout SCP (45%) > TAU (16%). Adverse events were adverse drug events (18%), which was only in SCP
Bruce [12]	SCP: 320 TAU: 278 %F: 69.1% in SCP, 74.5% in TAU	2y (study duration/ enrollment length 12 months)	Depression (older adults)	TAU	Suicidal ideation reduction (Scale for suicide ideation) SCP > TAU. Treatment response (HAMD-24 reduction ≥ 50%) SCP > TAU. Remission (HAMD-24 < 10) SCP > TAU	Dropout SCP (30.9%) = TAU (31.3%). Adverse events ND
Delgadillo [18]	SCP: 297 Stratified care: 505 %F: 65.1%	Up to 30 weeks	Depression (in patients with unipolar depression, post-traumatic stress disorder, obsessive–compulsive disorder, body dysmorphic disorder, phobias, and other anxiety disorders)	Stratified care	Stratified care was more effective in clinically significant improvement of depression symptoms (End point PHQ-9 ≤ 10 and PHQ-9 improvement ≥ 6 from baseline) than SCP	Dropout SCP (31%) = stratified care (31%). Adverse events ND
Guo [29]	SCP: 61 TAU: 59 %F: 64%	24w (study duration/ enrollment length)	MDD	TAU	Treatment response (HAMD-17 reduction ≥ 50%) and remission (HAMD-17 < 8) SCP > TAU	Dropout SCP (27.9%) = TAU (37.3%). Adverse events included anticholinergic side effects and did not differ between groups (49.2% in TAU, 39.3% in SCP)
Kronish [35]	SCP: 80 TAU: 77 %F: 54%	3y (study duration/ enrollment length)	Persistent depression and anxiety (in patients with acute coronary syndromes)	TAU	Anxiety improvement (HADS-A) compared to baseline in SCP, not in TAU, with significant correlation with depression symptoms (BDI)	Dropout SCP (25%) > TAU (8%). Adverse events not explicitly discussed in paper, but available in clinicaltrials.gov (NCT00158054). For serious adverse events, including ACS, chest pain, arrythmia, hyper/hypotension, shortness of breath, fatigue, CHF, death, other psych, suicidal ideation, and stroke SCP (27.5%), TAU (28.57%)
Ricken [51]	SCP: 74 TAU: 74 %F: 57% in SCP, 62% in TAU	3y (study duration/ enrollment length)	Depressive disorder	TAU	Remission (BRMS < 8) SCP = TAU	Dropout SCP (45%) > TAU (16%). Adverse events ND
Ricken [52]	SCP: 266 TAU: 84 %F: ND	Patient dependent	MDD	TAU	Remission (HAMD-21 < 10) SCP > TAU. Cost per remission in SCP < TAU	Dropout SCP (42%) > TAU (19%). Adverse events included side effects of medications (only in SCP, % not given)
Stoop [61]	SCP: 23 TAU: 23 %F: 56.5% in SCP, 46.5% in TAU	18 m (study duration/ enrollment length)	Anxiety and/or depression (in patients with diabetes/ asthma/ COPD)	TAU	Change in PHQ-9 score SCP = TAU	Dropout SCP = TAU (30% for both groups combined). Adverse events included cognitive symptoms which only happened to 1 patient in the study (group unknown)
Unutzer [70]	SCP: 906 TAU: 895 %F: 65%	1y (study duration/ enrollment length)	MDD, dysthymic disorder or both (older adults)	TAU	Treatment response (decrease in SCL-20 ≥ 50%) SCP > TAU	Dropout SCP (16%) = TAU (11%). Adverse events ND
**B. Non-randomized Controlled Trials**						
Kurian [37]	SCP: 32 TAU: 23 %F: 87%	1y (study duration/ enrollment length)	MDD	TAU	Symptom reduction (HAMD-17) SCP > TAU. Response (HAMD-17 reduction ≥ 50%) and remission (HAMD-17 < 8) SCP = TAU	Dropout SCP (19%) = TAU (17%). Adverse events ND
Sinyor [60]	SCP: 2876 %F: ND	4y (study duration/ enrollment length was largely patient dependent)	MDD	None (patients assigned to different treatments within each level of the SCP)	Remission (HAMD-17 < 8) rates: Level 1: 28% Level 2: 18–30% Level 3: 12–25% Level 4: 7–14%	Dropout 26%. Adverse events included inability to tolerate side effects (% ND)
					(QIDS-SR < 6) rates: Level 1: 37% Level 2: 56% Level 3: 62% Level 4: 67%	
**C. Cohort Studies**						
Awan [6]	SCP: 28 TAU: 92 %F: ND	16w (study duration/ enrollment length)	MDD with concurrent alcohol dependence	TAU	Symptom reduction (QIDS, BDI) and decrease in percent of heavy drinking days in SCP compared to baseline. No information on TAU group. Patient satisfaction SCP > TAU	Dropout SCP (46%) < TAU (78%). Adverse events ND
Emslie [21]	SCP depression: 24 SCP depression + ADHD: 15 TAU depression: 74 TAU depression + ADHD: 40 %F: 43%	4 m (study duration/ enrollment length)	Depression with or without ADHD (children and adolescents)	TAU	Symptom improvement (CGI) SCP > TAU. Treatment response (CGI score < 3) SCP > TAU	ND
Franx [26]	SCP: 400 TAU: 3956 %F: 70% in SCP, 64.9% in TAU	3y (study duration/ enrollment length)	Depression	TAU	Less antidepressants were prescribed in the SCP group than the TAU group	ND
Meeuwissen [42]	Data from adult patients in Dutch mental health care with mild, moderate or severe MDD was used, N and demographic information ND	ND	MDD	TAU	Using cost-utility analysis, SCP for mild, moderate-severe MDD is cost-effective compared to TAU with > 95% probability	ND
Pavuluri [49]	SCP: 17 TAU: 17 %F: ND	18 m (study duration/ enrollment length)	BD I (children and adolescents)	TAU	Treatment response (CGI – BP < 3) SCP > TAU	ND
Samokhvalov [56]	SCP: 81 TAU: 81 %F: 35%	16w (enrollment length/ study duration: Dec 2013–Dec 2015)	MDD with concurrent AUD	TAU	Symptom reduction (QIDS, BDI) in SCP compared to baseline. No information on TAU group. Alcohol consumption reduction SCP > TAU	Dropout SCP (19.5%) < TAU (69.1%). Adverse events ND
Suppes [62]	SCP: 141 TAU: 126 %F: 72% in SCP, 63% in TAU	12 m (study duration/ enrollment length)	BD I or schizoaffective disorder – BD type	TAU	Symptom reduction (BPRS, CARS-M, IDS-C) SCP > TAU	Dropout SCP (77%) = TAU (81%). Adverse events included medication side effects, which did not differ between SCP (48.9%) and TAU (42.1%)
Trivedi [66]	SCP: 175 TAU: 175 %F: 46%	12 m (study duration/ enrollment length)	MDD	TAU	Symptom reduction (IDS-C) SCP > TAU. Mental health improvement (SF-12) SCP > TAU	24.1% dropout overall. Adverse events ND

*Abbreviations*: *%F* Percent females, *AUD* Alcohol use disorder, *BDI* Beck depression inventory, *BPRS* Brief psychiatric rating scale, *BRMS* Bech-Rafaelson melancholia scale, *CGI* Clinical global improvement, *d* Days, *HADS-A* Hospital anxiety and depression scale—anxiety, *HAMD* Hamilton depression rating scale, *m* Months, *MADRS* Montgomery-Asberg Depression Rating Scale, *MCAS* Multnomah community ability scale, *MDD* Major depressive disorder, *MDE* Major depressive episode, *ND* Not described, *PACS* Penn alcohol craving scale, *PHQ* Patient health questionnaire, *QIDS-SR* Quick inventory of depressive symptomatology, self-report, *SCL* Symptom check list, *SCP* Structured care pathway, *TAU* Treatment as usual, *w* Weeks, *y* Years, *YMRS* Young mania rating scale

Of the 6 studies that reported remission rates, 4 studies reported higher remission rates in the SCP group compared to TAU [4, 12, 29, 52] whereas 2 studies reported no differences between SCP and TAU [7, 51]. Additionally, five studies also reported higher rates of treatment response in the SCP group than the TAU group [12, 20, 29, 35, 70] whereas one study reported that improvement in PHQ-9 scores did not differ between the SCP and TAU groups [61]. One study comparing SCP with stratified care reported greater clinical improvement as measured by PHQ-9 in stratified care [18]. There were no studies that reported higher remission or treatment response in the TAU group. Furthermore, a greater decline in suicidal ideation [4, 12] and anxiety [35], and lower cost per remission [52] were found with SCP treatment compared to TAU. Dropout rates ranged from 16 – 50% with SCP treatment, and 8 – 40% with TAU. Six studies reported higher rates of dropout in the SCP group [4, 7, 20, 35, 51, 52] and 5 studies reported no between-group differences [12, 18, 29, 39, 70] in dropout rate. Stoop and colleagues reported a combined dropout rate of 30% with no between-group differences [61]. Five studies mentioned adverse events, which included adverse drug events [7, 52] like anticholinergic side effects [29] or cognitive symptoms [61], and non-depression related psychiatric issues that were not specified in the study [35].

The Cochrane risk of bias tool [30] was used to assess the quality of the included RCTs, and is summarized in Table 3. Nine out of 11 studies used random sequence generation [7, 12, 18, 20, 29, 35, 51, 61, 70], while only 3 studies employed allocation concealment [20, 61, 70]. None of the RCTs had blinding of care-providers, while 6 studies had blinded outcome assessors [4, 20, 29, 35, 61, 70] and one study blinded participants [18]. For attrition bias, 6 studies had higher attrition in the SCP group than in TAU [7, 20, 35, 51, 52, 61] while 4 studies reported no between-group differences in attrition [4, 18, 29, 70]. One study reported a transient between-group difference in attrition rates, which became non-significant at the end of the study [12]. Intent to treat analysis (ITT) was performed by all but one study [61]. Selective reporting was only suspected in one study, where side effects were only mentioned for the SCP group, but not the TAU group [29]. Other potential sources of bias were examined with 4 studies having significant between-group differences in baseline demographic or clinical variables [4, 12, 29, 61]. One study did not provide demographic information of the participants [52], and another study did not statistically compare baseline characteristics between the groups [18].

**Table 3:** Q controlled trials assessed using the Cochrane risk of bias tool

**Author (year)**	**Bias**
Alexopoulos [4]	Selection bias (RSQ): ND
	Selection bias (AC): ND
	Performance bias: no blinding
	Detection bias: blinded assessors
	Attrition bias: No group difference. ITT done
	Reporting bias: none
	Other bias: baseline between-group differences in suicidal ideation
Bauer [7]	Selection bias (RSQ): Computer generated
	Selection bias (AC): ND
	Performance bias: no blinding
	Detection bias: no blinding
	Attrition bias: Higher attrition in SCP than TAU. ITT done
	Reporting bias: none
Bruce [12]	Selection bias (RSQ): Flip of Coin
	Selection bias (AC): ND
	Performance bias: ND
	Detection bias: ND
	Attrition bias: Transient group difference. ITT done
	Reporting bias: none
	Other bias: baseline between-group differences in suicidal ideation
Delgadillo [18]	Selection bias (RSQ): Computer generated
	Selection bias (AC): no blinding
	Performance bias: participants blinded to treatment group
	Detection bias: no blinding
	Attrition bias: No group difference. ITT done
	Reporting bias: none
	Other bias: statistical comparison for baseline characteristics between groups not reported
Guo [29]	Selection bias (RSQ): Table of random numbers
	Selection bias (AC): ND
	Performance bias: open label
	Detection bias: blinded assessors
	Attrition bias: No group difference. ITT done
	Reporting bias: side effects only detailed in the SCP group
	Other bias: patients in SCP younger than in TAU
Kronish [35]	Selection bias (RSQ): Computer generated
	Selection bias (AC): ND
	Performance bias: no blinding
	Detection bias: blinded assessors
	Attrition bias: Higher attrition in SCP than TAU. ITT done
	Reporting bias: none
Ricken [51]	Selection bias (RSQ): Computer generated
	Selection bias (AC): ND
	Performance bias: ND
	Detection bias: no blinding
	Attrition bias: Higher attrition in SCP than TAU. ITT done
	Reporting bias: none
Ricken [52]	Selection bias (RSQ): ND
	Selection bias (AC): ND
	Performance bias: ND
	Detection bias: ND
	Attrition bias: Higher attrition in SCP than TAU. ITT done
	Reporting bias: none
	Other bias: demographic information not included
Stoop [61]	Selection bias (RSQ): Computer generated
	Selection bias (AC): Sealed envelope
	Performance bias: ND
	Detection bias: outcomes rated by patient, practitioners blinded
	Attrition bias: Higher attrition in SCP than TAU. ITT not done
	Reporting bias: none
	Other bias: patients in SCP older than in TAU
Unutzer [70]	Selection bias (RSQ): Computer generated
	Selection bias (AC): Sealed envelope
	Performance bias: ND
	Detection bias: blinded assessors
	Attrition bias: No group difference. ITT done
	Reporting bias: none

*Abbreviations*: *AC* Allocation concealment, *ITT* Intention to treat, *ND* Not described, *RSQ* Random sequence generation, *SCP* Structured care pathway, *TAU* Treatment as usual

### Non-randomized clinical trials

Non-randomized controlled trials (NRCTs) are summarized in Table 2B. Both studies examined patients with MDD [37, 60]. TAU was used as a control group in one study [37], while the STAR*D trial compared treatment efficacy between different treatments within each level of the algorithm [60]. One study had a study length of 1 year [37], the other one a length of 4 years [60]. Both studies used HAMD-17 score less than 8 as the cutoff for remission [37, 60], and one study also used the quick inventory of depressive symptomatology, self-report (QIDS-SR) scale to measure cumulative remission rates [60]. Treatment response was also used as a main outcome in one study, where HAMD-17 score reduction of greater or equal to 50% was used as the cutoff [37].

In the review by Sinyor and colleagues, remission rates ranged from 18–30% in the first two levels of the algorithm, and decreased to 7–25% in the third and fourth levels when using the HAMD-17 [60]. The cumulative remission rates with the QIDS-SR were 37%, 56%, 62%, and 67%, for the four levels of the SCP, respectively [60]. Kurian and colleagues noted greater rate of treatment response in the SCP group compared to TAU, but did not find a between-group difference in rates of remission [37]. The dropout rate was 26% in the STAR*D trial, where adverse events included medication side effects [60]. Kurian and colleagues noted similar dropout rates between SCP and TAU but adverse events were not discussed [37].

### Cohort studies

Characteristics of cohort studies are summarized in Table 2C. Six of the 8 cohort studies examined patients with MDD or depression [6, 21, 26, 41, 56, 66], while the remainder examined patients with BD type I [49, 62]. TAU was used as control for all studies. The study length ranged from 4 months to 3 years. Improvement in symptoms was measured using the Quick inventory of depressive symptomatology scale (QIDS) and Beck depression inventory (BDI) together [6, 56], the Clinical global impression scale (CGI) [21], the CGI – bipolar scale (CGI-BP) [49], the Brief psychiatric rating scale (BPRS), the Clinician administered rating scale for mania (CARS-M), or the Inventory of depressive symptomatology scale – clinician administered (IDS-C) [62, 66]. Two studies used a CGI / CGI-BP score of less than 3 to classify treatment response [21, 49] reporting greater treatment response in the SCP group compared to TAU [21, 49]. Of the 5 studies measuring symptom reduction, 3 studies reported greater improvement with SCP treatment than TAU [21, 62, 66]. Two studies reported decreased symptoms in SCP but did not compare with TAU group [6, 56]. Four studies evaluated variables other than symptoms of depression or mania reporting greater decrease in alcohol consumption [56], greater patient satisfaction [6], lower antidepressant prescription rates [26], and higher cost-effectiveness [42] for the SCP group compared to TAU. Four studies reported dropout rates [6, 56, 62, 66], ranging from 19.5–77% in the SCP group and 69.1–81% in the TAU group with 2 studies reporting lower dropout rates in the SCP group compared to TAU [6, 56]. One study did not find a between-group difference in dropout rates [62]. Another study reported a dropout of 24.1% in both groups [66]. Adverse events were discussed in one study and included side effects of medications [62].

### Observational studies without a control group

Characteristics of observational studies without control groups are summarized in Table 4. Three of the fifteen studies examined patients with BD, type I or II [58, 63, 64], and the remaining twelve studies examined patients with MDD, depression, or other depressive disorders [1–3, 10, 11, 27, 41, 50, 57, 69, 72]. Study length ranged from 4 months to 5 years, with 2 studies being patient dependent [46, 63]. Seven studies measured rates of remission or recovery, using BRMS score of less than 6 [1], HAMD-17 score of less than 8 [10, 72], Montgomery-Asberg depression rating scale (MADRS) score of less than 8 [11, 50], BDI score of less than 11 [27], or Young mania rating scale score (YMRS) of less than 13 [58] as criteria for remission. Clinically meaningful treatment response or symptom improvement were also reported for 7 studies, i.e. change in BRMS score greater or equal to 50% [1], CGI global improvement subscale score greater than 2 [2], HAMD-17 score change greater or equal to 50% [3, 10, 72], MADRS score change greater or equal to 50% [11], YMRS score change greater or equal to 50% [58] as cutoffs. The Quick inventory of depressive symptomatology (QIDS-SR) was also used to assess symptoms of depression in one study [57].

**Table 4:** Observational studies without a control group

Author (year)	N/ %F	Duration (study duration and/or enrollment length)	Condition	Outcomes	Dropout and adverse events
Adli [1]	SCP: 119 %F: 33%	2y (study duration, enrollment length was patient dependent)	Depressive disorders	Remission (BRMS < 6) in 38%. Treatment response (BRMS change ≥ 50%) in 34%	Dropout 34%. Adverse events (17/119) included side effects
Agid [2]	SCP: 90 %F: 63%	8w (enrollment length, study duration unclear)	MDD	Symptom improvement (CGI global subscale > 2) in 49%	Dropout 18%. Adverse events ND
Akizuki [3]	SCP: 95 %F: 53%	4w (study duration/ enrollment length)	MDD (in patients with advanced cancer)	Algorithm applicable to 77% of patients. Symptom improvement (HAMD-17 reduction ≥ 50%) in 76% of patients	Dropout 23%. Adverse effects included antidepressant side effects (32%)
Birkenhager [10]	SCP: 203 %F: 68%	4y (study duration, enrollment length patient dependent)	MDD	Treatment response (HAMD-17 score reduction ≥ 50%) in 87%, Remission (HAMD-17 < 8) in 60%. Algorithm applicable to 50% of MDD patients	Dropout 16%. Adverse effects included side effects, worsening, or hypomania (N = 20)
Bondolfi [11]	SCP: 131 %F: 60%	4y (study duration, enrollment length patient dependent)	Depression	Remission (MADRS < 8) in 30.5%, treatment response (MADRS score reduction ≥ 50%) in 48.7%	Dropout 66%, Adverse effects in 21% contributing to dropout
Franx [27]	SCP: 514 %F: ND	6 m (study duration/ enrollment length)	Depression	Recovery (BDI < 11) in 30% of non-severe patients and 24% of severe patients	ND
Meeuwissen [41]	SCP: 28 %F: 61–62%	2.5y (study duration/ enrollment length)	MDD	96% adherence to treatment protocol. Decrease in percentage of patients being referred to secondary care	ND
Okamura [46]	SCP: 54 %F: 64%	Patient dependent (enrollment length)	MDD (in patients with advanced cancer)	Algorithm was applicable to 92% of patients	Dropout 35%. Adverse events included delirium and medication side effects (*N* = 2_
Ribeiz [50] – Duke somatic algorithm treatment for geriatric depression (STAGED)	SCP:67 %F: 78%	24w (study duration/ enrollment length)	MDD (older adults)	Remission (MADRS < 8) in 80.7%	Dropout 16%. Adverse events ND
Samokhvalov [57] – DA VINCI *This study has an overlapping sample of patients as Samokhvalov [56]	SCP:246 %F: 41.2%	SCP: 99.31 – 134.09 days (enrollment length, study duration: Dec 2013–Sep 2016)	MDD + AUD	70.7% of patients completed SCP, with significant reduction in number of drinking days per week, number of heavy drinking days per week, average number of standard drinks per drinking day, and weekly alcohol consumption. Significant decrease also in depression (QIDS-SR16) and cravings (PACS)	29.3% did not complete study. Adverse events ND
Scheffer [58] – adaptation of Systematic treatment enhancement program for bipolar disorder (STEP-BP)	SCP: 120 %F: ND	6 m (study duration/ enrollment length)	BDI or II in manic or mixed episode (refractory, children and adolescents)	Remission (YMRS < 13) in 75.8%. Treatment response (YMRS reduction ≥ 50%) in 74.2%	ND
Suppes [63]	SCP: 28 %F: 57%	Patient dependent, 5.2 m on average (enrollment length)	BDI or schizoaffective disorder – BD type	30% symptom improvement (BPRS or CGI) in 50% of patients	Dropout 18%. Adverse events included medication side effects (N ND)
Suppes [64]—TMAP	SCP: 69 %F: 67%	151d (enrollment length, study duration 1997–2000/2001)	BD I or schizoaffective disorder – BD type	Symptom improvement (BPRS) significant for both inpatients and outpatients. Improvement in community functioning (MCAS) only in inpatients, not outpatients	ND
Turner-Stokes [69]	SCP: 41 %F: 41%	15 m (study duration, enrollment length patient dependent)	Depression and concurrent severe complex disabilities (primarily brain injury)	Significant symptom improvement (BDI) post-treatment	Dropout 17%. Adverse events ND
Vermeiden [72]	SCP: 85 %F: 54%	5y (study duration, enrollment length patient dependent)	MDD	Remission (HAMD-17 < 8) in 46%, response (HAMD-17 score reduction ≥ 50%) in 71% of patients	Dropout 28% Adverse events included side effects, hypomanic conversion (*N* = 2), side effects (*N* = 4), and worsening of symptoms (*N* = 1)

*Abbreviations*: *%F* Percent females, *d* Days, *BDI* Beck depression inventory, *BPRS* Brief psychiatric rating scale, *BRMS* Bech-Rafaelson melancholia scale, *CGI* Clinical global improvement, *DA VINCI* Depression and alcoholism: validation of an integrated care initiative, *HAMD* Hamilton depression rating scale, *SCP* Structured care pathway, *m* Months, *MADRS* Montgomery-Asberg Depression Rating Scale, *MAOI* Monoamine oxidase inhibitor, *MCAS* Multnomah community ability scale, *MDD* Major depressive disorder, *ND* Not described, *PACS* Penn alcohol craving scale, *QIDS-SR16* Quick inventory of depressive symptomatology, *SCP* Structured care pathway, *TAU* Treatment as usual, *TCA* Tricyclic antidepressants, *w* weeks, *y* years, *YMRS* Young mania rating scale

Rates of remission ranged from 24 to 80.7%, and clinically meaningful symptom improvement or treatment response was found in 34 – 87% of patients with SCP treatment. One study noted a 30% symptom improvement in 50% of patients [63], and 3 studies reported significant improvement in symptoms after SCP treatment compared to baseline [57, 64, 69]. Studies examining the feasibility of SCPs reported that algorithms were applicable to 50–92% of the screened patients [3, 10, 46], completed by 70.7% [57], and adherence to the protocol was found in 96% of the patients and practitioners [41]. Furthermore, SCPs were found to improve community functioning [64] and decrease alcohol consumption and craving [57]. Eleven studies reported dropout rates ranging from 16–66% [1–3, 10, 11, 46, 50, 57, 63, 69, 72] and 7 studies discussed adverse events, which included known medication side effects [1, 3, 10, 11, 46, 63, 72], delirium [46], hypomanic switch [72], and worsening of symptoms [72].

## Discussion

MDD and BD are common mental health conditions associated with significant morbidity and mortality [22, 43, 44]. MDD and BD can be challenging to treat andlead to significant healthcare costs and burden to the patients [40, 43], especially if patients experience treatment resistance [22]. SCPs include treatment algorithms consisting of a series of steps and serve as recommendations and/or guidelines for applying evidence-based medicine and continuous monitoring of symptoms through validated outcome measures. This can result in a decrease of sub-standard variations in practice and better detection of non-response or clinical deterioration to improve patient care [5, 13].

In this systematic review, we examined the outcomes of SCPs in patients with MDD and BD with the aim of providing an ‘up-to-date’ overview of SCPs and assessment of their efficacy and effectiveness in individuals with mood disorders. This review provides an updated examination of SCPs in patients with MDD [8, 32, 71] and to our knowledge, is the first review to include SCPs for patients with BD.

Examining characteristics of included studies indicated that only 6 of the included studies examined SCPs for BD, of which one study was a RCT that was limited to patients with bipolar depression [7], and 2 cohort studies with a control group [49, 62], highlighting a clear need for more research to examine the effectiveness of SCPs in BD.

Fifteen of the studies included were observational studies without control groups and 11 were RCTs. While naturalistic studies allow studying treatments in a setting that is close to everyday clinical practice and provide valuable information on feasibility or effectiveness, well-designed RCTs are better positioned to assess treatment efficacy [45]. However, we included cohort studies and observational studies without control groups in this review to provide a broad picture and assessment of SCPs applied in different settings and study designs. It should be noted that the small number of RCTs examining SCPs in mood disorders show the need for more studies, particularly RCTs, to identify findings that are replicated in multiple studies adequately designed for this purpose.

We assessed the quality of the 11 RCTs included in this review using the Cochrane risk of bias tool [30]. Majority of the studies had random sequence generation to minimize selection bias, ITT analysis to minimize the effect of different rates of attrition between groups, and did not have selective reporting. Blinding of participants and care-providers would have been difficult given ethical and patient safety issues that are inherent to a clinical trial. In five studies, outcome assessors were not blinded, which would have been more feasible to include. It should also be noted that allocation concealment was only done in a minority of studies, however, in an open trial with participants and clinicians being aware of the treatment, this may not be as important. It is of note that half of the studies had other potential sources of biases, suggesting that while the RCTs were adequate in their quality, improvements can be made for developing future studies.

The SCPs found in the literature and included in this review were highly heterogeneous, not only with respect to the treatments and sequence of treatments in the algorithms but also in study length. In addition, there was heterogeneity in the scales used to assess symptom improvement, treatment response and remission, as well as heterogeneity in setting (i.e. primary care or tertiary hospitals), and study population for both clinical and demographic variables. Therefore, we performed a qualitative synthesis of the studies included in this review, as it would not be meaningful to quantitatively analyze and synthesize studies with largely varying designs and populations since effects can largely vary depending on setting (i.e. primary care vs. tertiary center) or medical and psychiatric comorbidities [8]. This heterogeneity had also been described in previously published reviews examining treatment algorithms [24, 32, 71]. This variation in treatment algorithms may be related to several aspects, including the fact that they were developed for different patient populations, such as those with comorbid psychiatric and non-psychiatric disorders, and for different care settings. Algorithms also likely differ as they were designed at different points in time with the earliest study published in 1998 [63] and the most recent in 2022 [18]. Evolving evidence and treatment guidelines over time [25, 34] as well as variation in treatment guidelines in different countries potentially contributed to the variation in SCP design. When the algorithms and/or designs were consistent between studies included in this review, this was because they belonged to the same project or used a previously published algorithm. Algorithms consistently applied across studies included those from TMAP [21, 37, 62, 64, 66], DA VINCI [6, 56, 57], IMPACT [20, 35, 70], GAP [7, 51, 52], and PROSPECT [4, 12]. One common pattern we observed was that ECT was offered in one of the last stages in most SCPs, despite previous studies demonstrating its treatment efficacy, cost-effectiveness and safety [19, 53].

With respect to clinical outcomes, more than half of the studies reported SCP treatment being superior to TAU in remission rates [4, 12, 29, 52], treatment response [12, 20, 21, 29, 35, 37, 49, 70], and change of symptom scores [21, 62, 66]. It is of note that no studies reported superiority of TAU over SCP for both MDD and BD, although one study reported superiority of stratified care compared to SCP [18]. SCP treatment was also found to be superior to TAU in decrease of suicidal ideation [4, 12], anxiety [35], alcohol consumption [56], and patient satisfaction [6]. When comparing to baseline (in the absence of a control group), SCP treatment was shown to decrease alcohol consumption [57] and improve community functioning [64] as well. Collectively, these findings indicate that SCPs may be more efficacious in treating depression and BD compared to TAU. However, the available evidence is mixed and inconsistent and more studies are required to clearly and comprehensively ascertain specific benefits of SCPs.

The heterogeneity of studies made it difficult to determine specific characteristics of the algorithms contributing to their effects. This was also mentioned in previous reviews of standardized treatment algorithms [24, 32]. Based on our inclusion criteria, the SCPs reviewed shared offering a structured algorithm that if properly adhered to ensured that patients received adequate trials of pharmacotherapy and/or psychotherapy and were closely monitored, and treatment was escalated when needed. These factors may have contributed to the favorable patient outcomes in SCPs. Indeed, previous studies have noted that the benefit of SCPs may primarily result from the structured protocols and mandatory assessment of treatment response rather than specific details of the algorithms [8, 32]. In this regard, several studies have examined the effect of measurement-based care, which focuses on using quantitative methods to monitor symptomatic improvement [29, 38, 59]. Also, a recent review noted that change in pharmacological agents did not affect the rate of remission after 2 antidepressant trials [8] in STAR*D, potentially suggesting that the structure of the SCPs including measurement-based care may be a main contributor to their effect, especially in patients with treatment resistant depression, who do not reach remission with 2 or more consecutive trials of antidepressants [55].

Four studies examined the feasibility of SCPs, where their algorithms were found to be applicable to the majority of the screened patients [3, 10, 46]. One study found that nearly all of those who received the SCP treatment were able to adhere to the treatment [41]. However, the majority of studies reported higher dropout rates in the SCP group compared to TAU [4, 7, 20, 35, 51, 52] and two studies showing lower dropout in the SCP group compared to TAU [6, 56]. Both studies with lower dropout in the SCP used the DA VINCI algorithm for treatment of patients with comorbid AUD and depression. Multiple factors of the DA VINCI algorithm may explain this success in decreasing dropout such as its multidisciplinary approach and the combined treatment of AUD and depression which may be more successful in retaining patients than treating either of these disorders. Overall, there was a large variation in dropout rates in both SCP and TAU groups. We were not able to identify specific and/or consistent factors related to study design or algorithm that were associated with dropout rates. Several studies have reported that medication side effects occurred as adverse events during the study [1, 3, 7, 10, 11, 46, 52, 60, 62, 63, 72]. SCPs often included medications with less tolerable side effects, including the thyroid hormone, MAOIs and mood stabilizers as monotherapy or as adjuncts in later algorithm steps, which may have contributed to the higher dropout rates. Furthermore, several studies had included medication dose increases as a step in the algorithm. This approach may have resulted in higher daily medication doses in SCPs, which in turn may have contributed to higher dropout rates secondary to adverse effects. With only one RCT not performing ITT analysis, it is difficult to ascertain how using ITT to account for dropout may affect overall results. It is of note that we did not find a consistent pattern of dropout rates and treatment response or symptomatic improvement. With respect to cost, it was found that SCP have higher cost effectiveness [42] and lower cost per remission [52] than TAU, supporting previous evidence suggesting that standardization of treatment steps and monitoring of treatment response can result in decreased cost of treatment [8].

This review has limitations that should be considered in the interpretation of its findings. It is important to note that SCPs can be defined differently from how they were defined in our study. While certain stepped care, collaborative care, and treatment algorithms fit our definition of SCPs, previous review papers using similar search terms have applied different selection criteria and thereby included different studies [24, 32, 71]. We further focused our review to studies designed with the aim of quantitatively evaluating the performance of a SCP, excluding studies that were designed for a different purpose, such as identifying biomarkers of treatment response. Also, we were mindful of the large heterogeneity in study design and interventions and decided to provide an updated summary rather than a quantitative synthesis (i.e., a meta-analysis). A meta-analysis examining the effectiveness of SCPs will be important in the future, especially to potentially inform clinical or policy recommendations, as more RCTs examining SCPs become available. In addition, our review included observational studies without control groups, which might limit the level of evidence presented. However, by evaluating a broad range of studies, including cohort studies, observational studies and RCTs, we were able to provide a broader assessment and overview of the application of SCPs in different settings. Also, there was a limited number of previous studies examining SCPs for patients with BD, however, accumulating evidence suggests potential effectiveness of SCPs in this population. More studies examining SCPs in patients with BD and MDD, especially with appropriate control groups, would be beneficial to further elucidate the effectiveness of SCPs and/or specific components of SCPs. Finally, we limited our search to 3 databases and only included peer-reviewed articles published in English, which limits the scope of this review. Future reviews performing a broader search of more databases may provide deeper insight into this topic.

## Conclusions

The findings of this systematic review suggest that SCPs are equally or more effective as TAU in the treatment of mood disorders. Evidence indicates that SCPs are potentially superior in certain settings, however, further studies are required to establish and confirm this, particularly for patients with BD, before specific recommendations can be made. Future studies should also specifically examine factors contributing to dropout and effectiveness to inform the development and implementation of more effective SCPs for patients suffering from mood disorders. In addition, identification of pragmatic clinical and biological markers to guide the use of SCPs may improve success and may inform integration of individualized medicine approaches and SCPs.

## Data Availability

Data are available from the corresponding author upon request.
